# IRLT: Integrating Reputation and Local Trust for Trustworthy Service Recommendation in Service-Oriented Social Networks

**DOI:** 10.1371/journal.pone.0151438

**Published:** 2016-03-10

**Authors:** Zhiquan Liu, Jianfeng Ma, Zhongyuan Jiang, Yinbin Miao, Cong Gao

**Affiliations:** 1 School of Computer Science and Technology, Xidian University, Xi’an, Shaanxi, China; 2 School of Cyber Engineering, Xidian University, Xi’an, Shaanxi, China; 3 School of Telecommunication Engineering, Xidian University, Xi’an, Shaanxi, China; Beihang University, CHINA

## Abstract

With the prevalence of Social Networks (SNs) and services, plenty of trust models for Trustworthy Service Recommendation (TSR) in Service-oriented SNs (S-SNs) have been proposed. The reputation-based schemes usually do not contain user preferences and are vulnerable to unfair rating attacks. Meanwhile, the local trust-based schemes generally have low reliability or even fail to work when the trust path is too long or does not exist. Thus it is beneficial to integrate them for TSR in S-SNs. This work improves the state-of-the-art Combining Global and Local Trust (CGLT) scheme and proposes a novel Integrating Reputation and Local Trust (IRLT) model which mainly includes four modules, namely Service Recommendation Interface (SRI) module, Local Trust-based Trust Evaluation (LTTE) module, Reputation-based Trust Evaluation (RTE) module and Aggregation Trust Evaluation (ATE) module. Besides, a synthetic S-SN based on the famous Advogato dataset is deployed and the well-known Discount Cumulative Gain (DCG) metric is employed to measure the service recommendation performance of our IRLT model with comparing to that of the excellent CGLT model. The results illustrate that our IRLT model is slightly superior to the CGLT model in honest environment and significantly outperforms the CGLT model in terms of the robustness against unfair rating attacks.

## Introduction

Nowadays, SNs are becoming increasingly prevalent and have affected many aspects of our daily life [1, 2]. For instance, we can make friends (e.g. www.facebook.com), post messages (e.g. www.twitter.com), go shoppings (e.g. www.amazon.com) and hunt for jobs (e.g. www.monster.com) via SNs. Moreover, the integration of SNs and services has become a trend and formed a new research area known as S-SN [3, 4].

**Fig 1:**
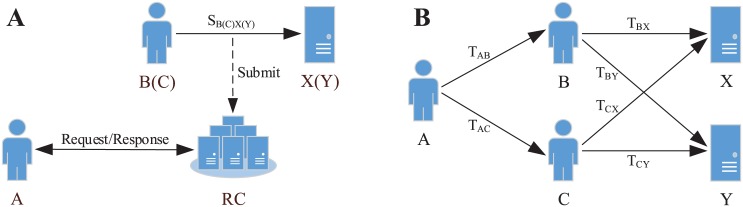
The classic TSR models in S-SNs. A: Reputation-based. B: Local trust-based.

Trust management plays a significant role in S-SNs as recommending the best services to service requesters is equivalent to recommending the most trustworthy services which meet their functional and personalized requirements [5, 6]. Recently, a large number of studies focus on adopting trust management as a solution for TSR in S-SNs [1, 3, 7–13], and the existing schemes are mainly divided into two categories, namely reputation-based and local trust-based TSR models:

- Reputation-based TSR models (see Fig 1A) generally contain a Reputation Center (RC) [14, 15] which is in charge of collecting the service ratings from all the service witnesses (i.e. the previous service consumers who have experienced the services, e.g. B and C) as well as calculating the trust values of services (e.g. X and Y). Furthermore, RC can derive the top-*k* recommendation list, which is usually independent of particular service requester (e.g. A), according to the trust values of services.
- Local trust-based TSR models (see Fig 1B) mainly depend on the trust propagation (i.e. partial transitivity of trust) in S-SNs [11, 16–18]. In many literatures [12, 13, 18, 19], S-SNs are modeled as directed graphs, in which nodes denote service consumers or services and directed edges represent the trust relationships among them. Multiple social trust paths may exist between a service requester (e.g. A) and a service (e.g. X or Y). For example, there exist two trust paths from A to X, namely A→B→X and A→C→X. Based on the social trust paths, service requester can evaluate the trust values of services and obtain the top-*k* recommendation list.

Though these classic reputation-based and local trust-based TSR models provide many brilliant ideas, there exist the following limitations in them:

- Reputation-based TSR models usually do not take user preference into consideration and merely provide a unique top-*k* recommendation list to all the service requesters. However, trust is subjective and different service requesters may have diverse user preferences [20]. Thus the reputation value of certain service is not always consistent with the opinion of individual service requester. Furthermore, this kind of schemes is usually vulnerable to unfair rating attacks, as malicious service providers are easy to collude with others to improve their own reputation values or slander their competitors [14, 21].
- Local trust-based TSR models calculate the local trust value of certain service based on the social trust paths from the active service requester to the service [17, 22]. This kind of schemes generally has low reliability when the trust path is too long as trust discounts along the trust path. More seriously, it may fail to work when there exists no available trust path between the active service requester and the service [3].

It is beneficial to integrate these two kinds of models for TSR in S-SNs as they have distinct characteristics: On the one hand, reputation-based TSR models can provide more general results than local trust-based ones as the former consider the opinions of all the service witnesses (including strange service witnesses, namely the ones without acceptable local trust relationships with the active service requester, and trustworthy ones) while the latter merely consider the opinions of trustworthy service witnesses. On the other hand, since the strange service witnesses have a higher likelihood of colluding with malicious service providers than the trustworthy ones and the service ratings from the former are more likely to be unfair, the service ratings in reputation-based trust evaluation should be filtered by the results of local trust-based trust evaluation for the purpose of easing unfair rating attacks.

However, to the best of our knowledge, there is no existing synthetical TSR model that comprehensively utilizes the above features. This is just the motivation of this work. In this paper, we improve the state-of-the-art CGLT scheme [3] and propose a novel IRLT model for TSR in S-SNs. The main features and contributions of our IRLT model are summarized as follows:

- Our IRLT model integrates reputation-based and local trust-based trust evaluations. In our IRLT model, we propose a comprehensive trust evaluation scheme which consists of four modules. In SRI module, a multiple Quality of Service (multi-QoS) based filtering method is proposed to select out all the candidate services which meet the functional requirements of the active service requester. In LTTE module, a Maximum Local Trust (MLT) evaluation algorithm is presented to identify all the trustworthy service witnesses and only the service ratings from them are considered in this module. In RTE module, the service ratings from all the service witnesses are considered with filtering by the outputs of LTTE module. Moreover, the preference similarity and rating number are viewed as two important weights. In ATE module, the outputs of RTE and LTTE modules are integrated by weighted summation to derive the finial trust values of candidate services and generate the top-*k* recommendation list.
- Our IRLT model has high service recommendation performance and strong robustness. In this work, we deploy a synthetic S-SN based on the famous Advogato (http://konect.uni-koblenz.de/networks/advogato) dataset and employ the classic DCG metric [23] to measure the service recommendation performance. In concrete terms, we conduct comprehensive experiments and analysis to compare the service recommendation performance of our IRLT model with that of the state-of-the-art CGLT model. The results illustrate that our IRLT model has slightly better performance than the CGLT model in honest environment and significantly outperforms the CGLT model in terms of the robustness against unfair rating attacks.

## Related Work

In recent years, TSR in S-SNs has been widely studied in both literatures and actual applications, and a large number of trust models have been proposed. In this section, we review some classic models according to the research approaches in them.

Reputation-based TSR models are widely employed in existing SNs, such as eBay (www.ebay.com), Yahoo (www.yahoo.com) and Epinions (www.epinions.com). Taking eBay for example [24, 25], it provide a simple reputation system which contains a centralized RC to manage all the service ratings (i.e. positive, neutral and negative). Feedback score (i.e. the subtraction between positive number and negative number) and positive feedback rate (i.e. the ratio between positive number and total number) are two important indicators of reputation, with which service requesters can make a better decision about service selection and avoid accessing to malicious services.

Recently, a vast number of schemes have been proposed to analyze and improve the performances of existing reputation systems [7–10]. Vavilis et al. [7] compared and classified lots of well-known reputation systems, as well as presented a novel reference model which can analyze and evaluate existing reputation systems and is also beneficial to the designs of new reputation systems. Wu et al. [8] proposed a novel Two-phase model for Calculating Service Reputation (TCSR). Dynamic weight formula and olfactory response formula are adopted in two phases, respectively. This model can provide more accurate reputation scores than the previous schemes. Xu et al. [9] presented a Reputation-enhanced QoS-based Service Discovery (RQSD) scheme through combining an extended Universal Description, Discovery and Integration (UDDI) registry, a reputation center and a discovery agent. This scheme takes user preference into consideration and can effectively match, rank and select services. Liu et al. [10] proposed a Fuzzy Logic-based Reputation (FLR) model for mitigating the adverse effects of unfair ratings. This model takes three factors (i.e. similarity, temporal and quantity) into consideration when calculating the weight of certain rating and greatly outperforms the previous schemes in terms of the robustness against unfair rating attacks.

Unlike the above reputation-based TSR models, some recent work focuses on evaluating trust in S-SNs from a local perspective [1, 11–13]. Kim et al. [11] proposed a novel Reinforcement Learning-based Trust Inference (RLTI) model for discovering the most reliable trust path and measuring trust level. They also evaluated the effects of the length of trust path and aggregation method on the accuracy of trust prediction. Liu et al. [12] presented a classic social network structure and transformed the optimal trust path selection issue into a Multi-Constrained Optimal Path (MCOP) selection problem which is NP-complete [26]. Furthermore, they also proposed a Monte Carlo method-based K-path (MONTE_K) selection algorithm to tackle this problem. Afterwards, they improved the previous scheme and presented a Heuristic Optimal Social Trust Path (H_OSTP) selection algorithm [13] which can find an approximate optimal trust path between any two nodes in S-SNs with a relatively low time complexity. Xu et al. [1] presented an efficient Preprocessing-based Search Strategy (PSS) for the purpose of accelerating trust path search by utilizing the structural properties of S-SNs. This scheme has similar search quality and lower time complexity when comparing to the previous approximation algorithms.

As mentioned in the introduction, reputation-based and local trust-based TSR models have different features, thus it is profitable to integrate them for TSR in S-SNs. Tang et al. [3] presented an outstanding CGLT model by combining global and local trust metrics. This hybrid model has better service recommendation performance than the mere global trust-based and local trust-based ones. However, it has two obvious limitations: a) Two kinds of trust evaluations are separately conducted without consideration of filtering unfair ratings. b) The user preferences on various service aspects (e.g. availability, security, price, response time, etc.) are also not taken into account. These shortages greatly limit the performance of CGLT model.

Aimming at integrating reputation-based and local trust-based trust evaluations for improving the service recommendation performance as well as overcoming the drawbacks of CGLT model, we propose a novel IRLT model in this paper and the intuitive comparisons with other TSR models are illustrated in Table 1.

**Table 1:** Intuitive comparisons between our IRLT model and other TSR models in S-SNs.

Trust models	Reputation-based	Local trust-based	Filtering unfair ratings	User preferences
eBay [24, 25]	√	×	×	×
TCSR [8]	√	×	√	×
RQSD [9]	√	×	×	√
FLR [10]	√	×	√	√
RLTI [11]	×	√	×	×
MONTE_K [12]	×	√	×	√
H_OSTP [13]	×	√	×	√
PSS [1]	×	√	×	√
CGLT [3]	√	√	×	×
IRLT	√	√	√	√

Note: √ and × denote support and non-support, respectively.

## Our IRLT Model and Trust Evaluation Method

In this section, we first briefly introduce the framework of our IRLT model. Next, we present the formal representations of elements in S-SNs. Moreover, we provide the detailed introductions of four modules.

### The framework of our IRLT model

**Fig 2:**
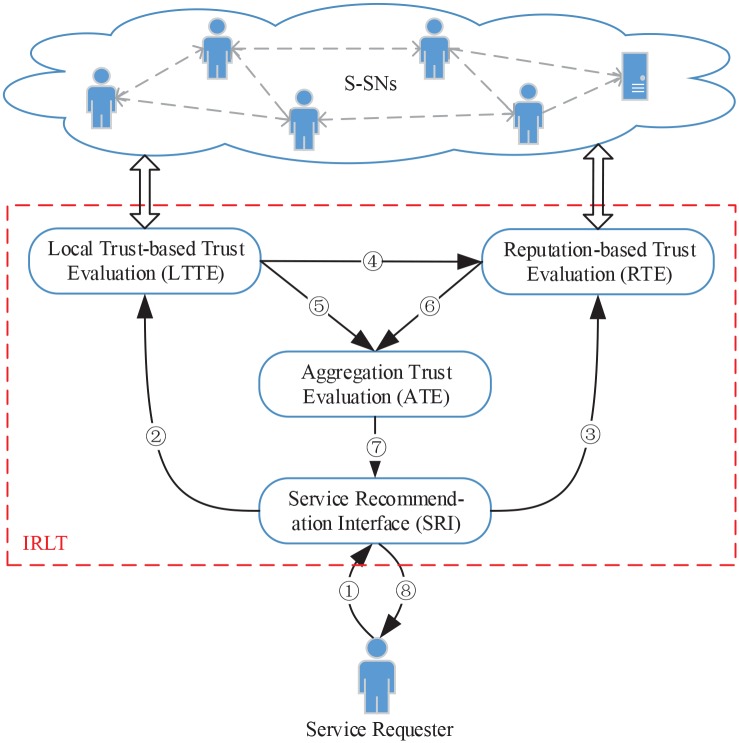
Our IRLT model for TSR in S-SNs. ①: TSR request. ② and ③: Candidate services. ④ and ⑤: Candidate services with local trust values. ⑥: Candidate services with reputation values. ⑦ and ⑧: Top-*k* recommendation list.

Fig 2 illustrates the framework of our IRLT model which mainly consists of four modules as follows:

- SRI module: This module is mainly in charge of receiving and pre-processing the requests from service requesters. When a service requester submits a request for TSR (as ①), SRI module first identifies all the candidate services which meet the functional requirements of the active service requester based on the multi-QoS filtering method, and then it sends requests to LTTE and RTE modules for evaluating the local trust values and reputation values of all the candidate services, respectively (as ② and ③).
- LTTE module: This module calculates the local trust values of candidate services merely based on the service ratings from trustworthy service witnesses. In this part, we proposed an efficient MLT algorithm to calculate the maximum local trust values of service witnesses and identify all the trustworthy service witnesses. The evaluation results of this module are then sent to RTE and ATE modules (as ④ and ⑤).
- RTE module: Different from LTTE module, this module mainly calculates the reputation values of candidate services based on the service ratings from all the service witnesses. The service ratings are filtered by the results of LTTE module for easing unfair rating attacks as the latter are more credible. Furthermore, the preference similarity and rating number are regarded as two important weights. The evaluation results of this module are also sent to ATE module (as ⑥).
- ATE module: This module takes charge of integrating the evaluation results of LTTE and RTE modules by weighted summation and obtaining the final trust values of all the candidate services. Afterwards, this module generates the top-*k* recommendation list and sends it to the active service requester via SRI module (as ⑦ and ⑧).

### The formal representations of elements in S-SNs

For illustration purposes, we give the following formal representations of elements in S-SNs:

- *M*: The number of service consumers.
- *N*: The number of services.
- *A*: The number of service aspects.
- *SC*(*i*): Service consumer *i*, where 1 ≤ *i* ≤ *M*.
- *SC*: The set of service consumers, i.e. *SC* = {*SC*(*i*)}, where 1 ≤ *i* ≤ *M*.
- *SR*(*j*): Service *j*, where 1 ≤ *j* ≤ *N*.
- *SR*: The set of services, i.e. *SR* = {*SR*(*j*)}, where 1 ≤ *j* ≤ *N*.
- *TR*(*i*, *i*′): The trust value of *SC*(*i*) to *SC*(*i*′), where 1 ≤ *i*, *i*′ ≤ *M* and *i* ≠ *i*′. It is denoted as a real number within the range of [0, 1]. A larger number means a stronger trust relationship and vice versa.
- *TR*: The set of trust values among service consumers, i.e. *TR* = {*TR*(*i*, *i*′)}, where 1 ≤ *i*, *i*′ ≤ *M* and *i* ≠ *i*′.
- *TS*(*i*): The set of trustworthy service witnesses in the view of *SC*(*i*), i.e. *TS*(*i*) = {*SC*(*i*′)}, where 1 ≤ *i*, *i*′ ≤ *M* and service witness *SC*(*i*′) is trustworthy from the perspective of *SC*(*i*).
- *SS*(*j*): The service witness set of *SR*(*j*), i.e. *SS*(*j*) = {*SC*(*i*′)}, where 1 ≤ *i*′ ≤ *M*, 1 ≤ *j* ≤ *N* and *SC*(*i*′) has consumed and rated for *SR*(*j*).
- *FS*(*j*): The fair service witness set of *SR*(*j*), i.e. *FS*(*j*) = {*SC*(*i*′)} ⊂ *SS*(*j*), where 1 ≤ *i*′ ≤ *M*, 1 ≤ *j* ≤ *N* and *SC*(*i*′) has consumed and given a fair rating to *SR*(*j*).
- *RT*(*i*, *j*, *a*): The rating value of *SC*(*i*) to *SR*(*j*) on service aspect *a*, where 1 ≤ *i* ≤ *M*, 1 ≤ *j* ≤ *N* and 1 ≤ *a* ≤ *A*. It is represented as an integer between 0 and 4, indicating very dissatisfied, dissatisfied, general, satisfied and very satisfied, respectively.
- ***RT***(*i*, *j*): The rating vector of *SC*(*i*) to *SR*(*j*), i.e. ***RT***(*i*, *j*) = (*RT*(*i*, *j*, 1), *RT*(*i*, *j*, 2), …, *RT*(*i*, *j*, *A*)), where 1 ≤ *i* ≤ *M* and 1 ≤ *j* ≤ *N*.
- *RT*: The set of rating vectors, i.e. *RT* = {***RT***(*i*, *j*)}, where 1 ≤ *i* ≤ *M* and 1 ≤ *j* ≤ *N*.
- *PR*(*i*, *a*): The preference weight value of *SC*(*i*) on service aspect *a*, where 1 ≤ *i* ≤ *M*, 1 ≤ *a* ≤ *A* and $\sum_{a=1}^{A}PR\left(i,a\right)\neq 0$. It is denoted as an integer between 0 and 2, meaning uninterested, general and very interested, respectively.
- ***PR***(*i*): The preference weight vector of *SC*(*i*), i.e. ***PR***(*i*) = (*PR*(*i*, 1), *PR*(*i*, 2), …, *PR*(*i*, *A*)), where 1 ≤ *i* ≤ *M*.
- *PR*: The set of preference weight vectors, i.e. *PR* = {***PR***(*i*)}, where 1 ≤ *i* ≤ *M*.
- *RS*(*i*, *j*): The synthetical rating score of *SC*(*i*) to *SR*(*j*), i.e. $RS\left(i,j\right)=\frac{\sum_{a=1}^{A}RT\left(i,j,a\right)*PR\left(i,a\right)}{4*\sum_{a=1}^{A}PR\left(i,a\right)}$, where 1 ≤ *i* ≤ *M* and 1 ≤ *j* ≤ *N*.
- *SN*: The social network which is composed of all the service consumers and the trust relationships among them, i.e. *SN* = (*SC*, *TR*).
- *BN*: The bipartite network which is made up of all the service consumers and services as well as the service ratings and preference weights, i.e. *BN* = (*SC*, *SR*, *RT*, *PR*).

S-SN can be viewed as a combination of *SN* and *BN*. To balance computational complexity and evaluation performance, the trust (i.e. *TR*(*i*, *i*′)) and service rating (i.e. *RT*(*i*, *j*, *a*)) are represented as different forms. Specifically, the former is denoted as a real number in order to reduce the computational complexity of LTTE module, while the latter is denoted as a vector including the rating values on various service aspects with different user preference weights so as to improve the evaluation performance of RTE module.

### Service recommendation interface

As we mentioned earlier, this module mainly takes charge of selecting out all the candidate services which satisfy the functional requirements of the active service requester. To this end, we propose a multi-QoS based filtering method and a simple example is shown in Table 2.

**Table 2:** A simple example of our multi-QoS based filtering method.

Entities	Functions	Prices	Response times	Satisfy or not
*SC*(1)	*Datastorage*	≤ $1000	≤0.5*s*	-
*SR*(1)	*Dataencryption*	$500	0.2*s*	×
*SR*(2)	*Datastorage*	$1500	0.2*s*	×
*SR*(3)	*Datastorage*	$500	0.7*s*	×
*SR*(4)	*Datastorage*	$500	0.2*s*	√
*SR*(5)	*Datastorage*	$1000	0.5*s*	√

Note: √ and × denote satisfaction and dissatisfaction, respectively.

When *SC*(1) submits a request which contains its multi-QoS requirements (i.e. Function is “*Datastorage*”, Price≤“$1000” and Response time ≤ “0.5*s*”), every service in *SR* = {*SR*(1), *SR*(2), …, *SR*(5)} is checked whether meets the multi-QoS needs of *SC*(1) or not. We can easily find that *SR*(1), *SR*(2) and *SR*(3) do not satisfy the “Function”, “Price” and “Response time” requirements of *SC*(1), respectively, so they are filtered out. While both *SR*(4) and *SR*(5) meet the multi-QoS needs of *SC*(1), thus they are candidate services and are then sent to RTE and LTTE modules for trust evaluations.

### Local trust-based trust evaluation

When evaluating the local trust value of certain candidate service, only the service ratings from trustworthy service witnesses are considered, while the opinions of the other service witnesses are ignored. For the purpose of identifying all the trustworthy service witnesses, we propose an efficient MLT algorithm (i.e. Algorithm 1) to obtain the maximum local trust values of all the service witnesses.

As we know, S-SNs have the “small-world” characteristic [12, 27]. Moreover, the trust will decay with the increasing trust path length and the evaluation reliability will be very low when the trust path is too long [3]. Thus we take the hop into account in our MLT algorithm and a service witness who can merely be reached via a very long trust path (i.e. its hop is bigger than the maximum allowable hop *H*) is considered untrustworthy. Suppose *SC*(*p*_0_)→*SC*(*p*_1_)→…→*SC*(*p*_*t*_) is the optimal trust path from *SC*(*i*) to *SC*(*i*′) (where *SC*(*p*_0_) = *SC*(*i*) and *SC*(*p*_*t*_) = *SC*(*i*′)), then the maximum local trust value *MT*(*i*, *i*′) (i.e. *MT*[*i*′] in Algorithm 1) of *SC*(*i*′) in the perspective of *SC*(*i*) can be derived from [18]:

$$\begin{array}{c} MT\left(i,i^{\prime }\right)=\{\begin{array}{cc} \frac{\prod_{m=0}^{t-1}TR(p_{m},p_{m+1})}{t^{\alpha }}, & \;if\;t\leq H,\\ 0, & \;otherwise. \end{array} \end{array}$$ (1)

Where *t* is the hop of trust path from *SC*(*i*) to *SC*(*i*′) and *α* is a constant (e.g. 0.5) which controls the speed of trust decay. If *MT*(*i*, *i*′) reaches the trust threshold *TH*(*i*) of *SC*(*i*), *SC*(*i*′) is considered trustworthy (i.e. *SC*(*i*′)∈*TS*(*i*)) and vise versa. Similarly, we can obtain all the elements of *TS*(*i*) and then calculate the local trust *LT*(*i*, *j*) of service *SR*(*j*) in the view of *SC*(*i*) as [11]:

$$\begin{array}{c} LT\left(i,j\right)=\{\begin{array}{cc} \frac{\sum_{SC\left(i^{\prime }\right)\in TS\left(i\right)\cap SS\left(j\right)}RS\left(i^{\prime },j\right)*MT\left(i,i^{\prime }\right)}{\sum_{SC\left(i^{\prime }\right)\in TS\left(i\right)\cap SS\left(j\right)}MT\left(i,i^{\prime }\right)}, & \;if\;TS(i)\cap SS(j)\neq ⌀,\\ \mu , & \;otherwise. \end{array} \end{array}$$ (2)

Where *μ* is a default local trust value in the range of [0, 1] (e.g. 0.1). We can find that both *MT*(*i*, *i*′) and *LT*(*i*, *j*) range from 0 to 1. From Algorithm 1, Eqs (1) and (2), we can obtain the local trust values of all the candidate services which are then sent to RTE and ATE modules for further processing.

**Algorithm 1 Our MLT algorithm**

**Input**: *SN* = (*SC*, *TR*), *H*, *SC*(*i*), *α*; /* Social network, maximum allowable hop, active service requester and trust decay parameter, respectively. */

**Output**: *MT*; /* Maximum local trust values of all the service consumers in SC from the perspective of *SC*(*i*). */

1: *VS* ⇐ ⌀; /* The visited service consumer set which is initialized to an empty set. */

2: *MT*, *HP*; /* The arrays of maximum local trust values and corresponding hops from *SC*(*i*), respectively. */

3: *MT*[*i*] ⇐ 1;

4: *HP*[*i*] ⇐ 0;

5: **for** each *SC*(*i*′) ∈ *SC* − {*SC*(*i*)} **do**

6:  **if** *TR*(*i*, *i*′) > 0 **then**

7:   *MT*[*i*′] ⇐ *TR*(*i*, *i*′);

8:   *HP*[*i*′] ⇐ 1;

9:  **else**

10:   *MT*[*i*′] ⇐ 0;

11:   *HP*[*i*′] ⇐ ∞;

12:  **end if**

13: **end for**

14: Add *SC*(*i*) to *VS*;

15: **while** |*VS*| < |*SC*| **do**

16:  *SC*(*i*′) ⇐ Find the service consumer with the maximum local trust value in *SC* − *VS*;

17:  **if** *HP*[*i*′] < *H* **then**

18:   **for** each *SC*(*i*′′) ∈ *SC* − *VS* − {*SC*(*i*′)} **do**

19:    **if** *TR*(*i*′, *i*′′) > 0 and $MT\left[i^{\prime }\right]*TR\left(i^{\prime },i^{\prime \prime }\right)*{\left(\frac{HP[i^{\prime }]}{HP[i^{\prime }]+1}\right)}^{\alpha }>MT\left[i^{\prime \prime }\right]$ **then**

20:     $MT\left[i^{\prime \prime }\right]⇐MT\left[i^{\prime }\right]*TR\left(i^{\prime },i^{\prime \prime }\right)*{\left(\frac{HP[i^{\prime }]}{HP[i^{\prime }]+1}\right)}^{\alpha }$;

21:     *HP*[*i*′′] ⇐ *HP*[*i*′] + 1;

22:    **end if**

23:   **end for**

24:  **end if**

25:  Add *SC*(*i*′) to *VS*;

26: **end while**

27: **return** *MT*;

### Reputation-based trust evaluation

Next, we present how to calculate the reputation values of candidate services. In many reputation systems of existing e-commerce websites, the reputation value of centain service is merely a simple average of all the rating values given to it. Taking Tmall (www.tmall.com) for example, it is a well-known e-commerce website and the service rating consists of five grades, namely very dissatisfied, dissatisfied, general, satisfied and very satisfied, which are denoted as 1 ∼ 5, respectively. It also considers various service aspects, namely service performance, service attitude, delivery speed and logistics speed. Though it is intuitive and beneficial to potential service consumers, it is vulnerable to unfair rating attacks and neither user preference nor rating number is taken into account when calculating the reputation value of certain service.

To improve the evaluation performance, we modify the reputation calculation method used in Tmall. In concert terms, we first filter unfair ratings according to the results of LTTE module as the latter are more credible in the view of service requester. If the distance between rating score *RS*(*i*′, *j*) and local trust value *LT*(*i*, *j*) exceeds system threshold *λ* (e.g. 0.4), then *RS*(*i*′, *j*) is filtered out as it is considered unfair (i.e. service witness *SC*(*i*′)∉*FS*(*j*)) and vise versa. Furthermore, we also take the following two weights into consideration:

- User preference weight: In the perspective of service requester *SC*(*i*), the rating score *RS*(*i*′, *j*) from service witness *SC*(*i*′) is more credible if *SC*(*i*) and *SC*(*i*′) have more similar preferences and vice versa. Thus we define the user preference weight *GP*(*i*, *i*′) based on the weighted Euclidean distance between ***PR***(*i*) and ***PR***(*i*′) [6]:
  $$\begin{array}{ccc} GP(i,i^{\prime }) & = & 1-\frac{1}{2}*{∥\text{PR}\left(i\right)-\text{PR}\left(i^{\prime }\right)∥}_{Euclidean}\\ & = & 1-\frac{1}{2}*\sqrt{\frac{\sum_{a=1}^{A}{\left(PR\left(i,a\right)-PR\left(i^{\prime },a\right)\right)}^{2}*PR\left(i,a\right)}{\sum_{a=1}^{A}PR\left(i,a\right)}}. \end{array}$$ (3)
- Rating number weight: As we know, the rating number of service also affects its reputation value. Intuitively, the reputation value of service *SR*(*j*) is higher if its fair rating number |*FS*(*j*)| is larger and vice versa. So we define the rating number weight *GN*(*j*) of *SR*(*j*) as a piecewise function of |*FS*(*j*)|:
  $$\begin{array}{c} GN\left(j\right)=\{\begin{array}{cc} \frac{1}{2}*{\left(\frac{\left|FS(j)\right|}{AV}\right)}^{2}, & \;if\;|FS(j)|\leq AV,\\ \frac{1}{2}*\left(\sqrt{\frac{|FS(j)|-AV}{MA-AV}}+1\right), & \;otherwise, \end{array} \end{array}$$ (4)
  where *AV* and *MA* are the average and maximum fair rating numbers of candidate services, respectively. In general, *GN*(*j*) grows from 0 to 1 with the increase of |*FS*(*j*)| from 0 to *MA*, and the growth speed of *GN*(*j*) when |*FS*(*j*)| ≤ *AV* is higher than that when *AV* < |*FS*(*j*)| ≤ *MA*. Specifically, the value of *GN*(*j*) is 0, 0.5, 1 when |*FS*(*j*)| equals to 0, *AV*, *MA*, respectively.

Combining *GP*(*i*, *i*′) and *GN*(*j*), the reputation value *RP*(*i*, *j*) of *SR*(*j*) in the sight of *SC*(*i*) can be derived from

$$\begin{array}{c} RP\left(i,j\right)=\{\begin{array}{cc} \frac{\sum_{SC\left(i^{\prime }\right)\in FS\left(j\right)}RS\left(i^{\prime },j\right)*GP\left(i,i^{\prime }\right)}{\sum_{SC\left(i^{\prime }\right)\in FS\left(j\right)}GP\left(i,i^{\prime }\right)}*GN\left(j\right), & \;if\;FS(j)\neq ⌀,\\ \nu , & \;otherwise. \end{array} \end{array}$$ (5)

Where *ν* is a default reputation value in the range of [0, 1] (e.g. 0.1). We can easily find that *GP*(*i*, *i*′), *GN*(*j*) and *RP*(*i*, *j*) range from 0 to 1 due to the normalization processing. The above calculation process (i.e. Eqs (3) ∼ (5)) is run for all the candidate services to obtain their reputation values which are then also sent to ATE module for further processing.

### Aggregation trust evaluation

As mentioned earlier, this module is mainly in charge of integrating the evaluation results of LTTE and RTE modules by weighted summation and obtaining the final trust values of all the candidate services. Specifically, the final trust value *FT*(*i*, *j*) of *SR*(*j*) in the sight of *SC*(*i*) can be gained from

$$\begin{array}{c} FT(i,j)=\omega *LT(i,j)+(1-\omega )*RP(i,j). \end{array}$$ (6)

Where *ω* is a parameter which ranges from 0 to 1 and controls the weights of LTTE and RTE modules in aggregation trust evaluation, thus we can easily find that the range of *FT*(*i*, *j*) is also [0, 1]. Afterwards, this module can generate the top-*k* (where *k* is a parameter) recommendation list according to the final trust values of candidate services and sends it to *SC*(*i*) via SRI module. A simple example is shown in Table 3.

**Table 3:** A simple example of aggregation trust evaluation.

Cases	*SR*(1)	*SR*(2)	*SR*(3)	*SR*(4)	*SR*(5)	*RL*^*a*^ (*k* = 3)^*b*^	*RL* (*k* = 5)
*LT*	0.75	0.20	0.70	0.25	0.40	-	-
*RP*	0.65	0.30	0.35	0.60	0.45	-	-
*FT* (*ω* = 0)	0.65	0.30	0.35	0.60	0.45	1 ≺^*c*^ 4 ≺ 5	1 ≺ 4 ≺ 5 ≺ 3 ≺ 2
*FT* (*ω* = 0.25)	0.68	0.28	0.44	0.51	0.44	1 ≺ 4 ≺ 3	1 ≺ 4 ≺ 3 ≺ 5 ≺ 2
*FT* (*ω* = 0.5)	0.70	0.25	0.53	0.43	0.43	1 ≺ 3 ≺ 5	1 ≺ 3 ≺ 5 ≺ 4 ≺ 2
*FT* (*ω* = 0.75)	0.73	0.23	0.61	0.34	0.41	1 ≺ 3 ≺ 5	1 ≺ 3 ≺ 5 ≺ 4 ≺ 2
*FT* (*ω* = 1)	0.75	0.20	0.70	0.25	0.40	1 ≺ 3 ≺ 5	1 ≺ 3 ≺ 5 ≺ 4 ≺ 2

^*a*^RL is short for recommendation list.

^b^The top-*k* recommendation lists when *k* = 1, 2 and 4 are omitted due to space limitation.

^*c*^ ≺ denotes that the former is prior to the latter in the top-*k* recommendation list.

*SR*(1)∼*SR*(5) are five candidate services with diverse local trust and reputation values (i.e. *LT* and *RP*, respectively). Their final trust values (i.e. *FT*) and top-*k* recommendation lists (i.e. *RL*) change with *ω* as illustrated in Table 3. Specifically, when *ω* equals to 0 or 1, *FT* is equivalent to *RP* or *LT*, respectively. In other cases, *FT* falls between *LT* and *RP*. If two candidate services have the same *FT*, the service with larger *LT* should be prior to the other one in the top-*k* recommendation list (as local trust-based evaluation (*LT*) is more credible than reputation-based evaluation (*RP*) in the view of service requester).

## Experiments and Analysis

To illustrate the performance of our IRLT model, we present a series of experiments and analysis in this section. In concrete terms, we deploy a synthetic S-SN based on the famous Advogato dataset, and employ the well-known DCG metric to measure the service recommendation performance of our IRLT model through comparing to the state-of-the-art CGLT model in honest environment and malicious environment with unfair rating attacks.

### Experiment settings

In this work, the comprehensive experiments are implemented through Java language on a Linux server with 2.83GHz × 4 CPU and 8G RAM. Specifically, we deploy a synthetic S-SN which contains a social network SN and a bipartite network BN. In SN part, we adopt Advogato which is a famous trust network dataset consisting of 6541 nodes (i.e. service consumers) and 51127 directed edges (i.e. trust relationships among service consumers) as well as containing three kinds of different trust relationships (i.e. apprentice, journeyer and master, of which corresponding trust values are 0.6, 0.8 and 1.0, respectively). In BN part, we assume that there exist 50 candidate services and each service has 100 random service witnesses in SN. Each service witness provides a service rating to corresponding service and the preference weights of service consumers in SN are randomly generated. The parameters in experiments are set as illustrated in Table 4.

**Table 4:** The values of parameters in experiments.

Parameters	Descriptions	Values
*M*	The number of service consumers	6541
*N*	The number of candidate services	50
*A*	The number of service aspects	3
*H*^*a*^	The maximum allowable hop in Algorithm 1	7
*α*	The trust decay parameter in Algorithm 1	0.5
*μ*	The default local trust value in Eq (2)	0.1
*ν*	The default reputation value in Eq (5)	0.1
*ω*	The weight parameter in Eq (6)	0.5

^a^As we know, S-SNs have the “small-world” characteristic [12, 27] and most of nodes can be reached within 6 hops. As with some classic schemes [12, 18], *H* is also set to 7 (slightly bigger than 6) in order to make our IRLT model more practical.

### Experiment 1

In this part, we mainly illustrate the service recommendation performance of our IRLT model in honest environment (i.e. all the service witnesses are honest and provide fair service ratings to corresponding services) through comparing to the state-of-the-art CGLT model. As with the outstanding CGLT scheme, we adopt the well-known DCG metric to measure the service recommendation performance. Specifically, we define three kinds of DCG metrics (i.e. Eqs (7) ∼ (9)) to evaluate the performance of the top-*k* recommendation list in terms of local trust value (i.e. *LT*), reputation value (i.e. *RP*) and final trust value (i.e. *FT*), respectively.

$$\begin{array}{c} DCG\;-\;LT\left(i,k\right)=\sum_{s=1}^{k}\frac{2^{LT(i,j(s))}-1}{log_{2}\left(1+s\right)}, \end{array}$$ (7)

$$\begin{array}{c} DCG\;-\;RP\left(i,k\right)=\sum_{s=1}^{k}\frac{2^{RP(i,j(s))}-1}{log_{2}\left(1+s\right)}, \end{array}$$ (8)

$$\begin{array}{c} DCG\;-\;FT\left(i,k\right)=\sum_{s=1}^{k}\frac{2^{FT(i,j(s))}-1}{log_{2}\left(1+s\right)}. \end{array}$$ (9)

**Fig 3:**
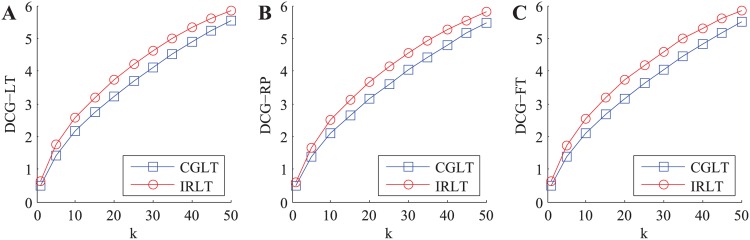
The variations of three kinds of DCG metric values with *k* in honest environment. A: DCG-LT. B: DCG-RP. C: DCG-FT.

Where *k* is the size of recommendation list, and *LT*(*i*, *j*(*s*)), *RP*(*i*, *j*(*s*)) and *FT*(*i*, *j*(*s*)) are the local trust value, reputation value and final trust value of the *s*-th service in the top-*k* recommendation list (in the opinion of *SC*(*i*)), respectively. In each experiment, we randomly choose a service requester from SN and generate the top-*k* recommendation list for it through utilizing the CGLT and our IRLT schemes, respectively. Afterwards, we can calculate three kinds of DCG metric values in the CGLT and our IRLT schemes when *k* takes different values (i.e. 1 ∼ 50), respectively. The experiment is repeated 5000 times and the average outputs are illustrated in Fig 3.

From Fig 3, we can easily find that three kinds of DCG metric values in two kinds of trust models continually increase with *k*, and three kinds of DCG metric values in our IRLT model are slightly higher than those in the CGLT model, respectively. This indicates that the top-*k* services obtained from our IRLT model have higher local trust values, reputation values and final trust values than those derived from the CGLT model, thus our IRLT model has a little better service recommendation quality than the state-of-the-art CGLT model in honest environment.

### Experiment 2

In this part, we focus on analyzing and validating the robustness of our IRLT model against the following two kinds of unfair rating attacks [28, 29]:

- Ballot stuffing: Malicious service providers collude with service consumers to raise their own trust values.
- Bad mouthing: Malicious service providers collude with service consumers to slander their honest competitors.

As we know, these two kinds of unfair ratings will bring risk to service requesters, thus an excellent TSR scheme should be able to detect and filter them out. In the outstanding CGLT model, two kinds of trust evaluations are separately made without consideration of filtering unfair ratings. While in our IRLT model, we notice that the strange service witnesses have a higher likelihood of colluding with malicious service providers than the trustworthy ones and the service ratings from the former are more likely to be unfair, thus we filter the service ratings in RTE module by utilizing the results from LTTE module. As a result, our IRLT should have stronger robustness against these two kinds of unfair rating attacks. Next we validate the above analysis through three series of experiments, in which the service ratings from trustworthy service witnesses are assumed to be fair and 50 candidate services are composed of 25 honest services and 25 malicious services.

#### Ballot Stuffing

**Fig 4:**
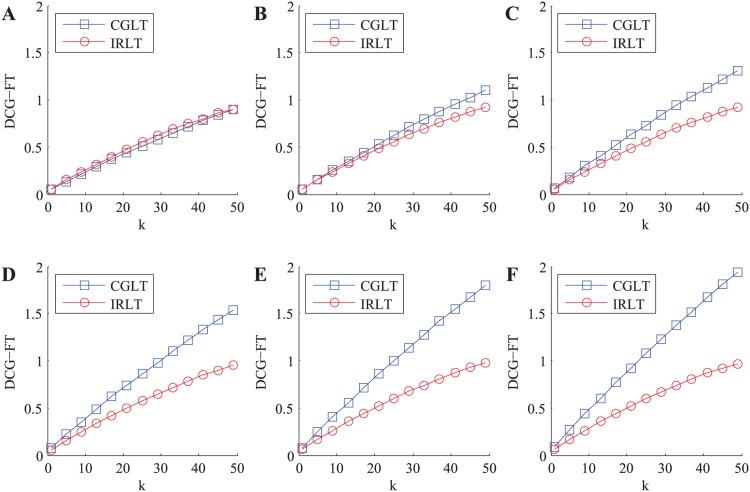
The variation of total DCG-FT metric value of malicious services with *k* in malicious environment with ballot stuffing attack. A: PCSC = 0%. B: PCSC = 20%. C: PCSC = 40%. D: PCSC = 60%. E: PCSC = 80%. F: PCSC = 100%.

In this part, we mainly validate the robustness of our IRLT model against ballot stuffing attack through comparing to the outstanding CGLT model. In the ballot stuffing attack, the collusive service consumers provide high rating scores to malicious services in spite of their poor performances. In each experiment, we vary the Percentage of Collusive Service Consumers (PCSC) and calculate the three kinds of metric values of malicious services in the top-*k* recommendation list in each case, respectively. The experiment is repeated 5000 times and the average outputs of DCG-FT metric are shown in Fig 4 (The variation curves of DCG-LT and DCG-RP metrics are omitted due to space limitation, and the same below).

In the ideal case (i.e. PCSC = 0%, see Fig 4A), the variation curve of total DCG-FT metric value of malicious services in our IRLT model is approximately consistent with that in the CGLT model. With the increase of PCSC, the variation curve in the CGLT model gets steeper and steeper while that in our IRLT model keeps almost unchanged, so the gap of two variation curves gradually grows. In the extreme case (i.e. PCSC = 100%, see Fig 4F), the gap of two variation curves reaches the maximum amount and the total DCG-FT metric value of malicious services in our IRLT model is significantly lower than that in the CGLT model.

It is obvious that malicious services mean risk for service requesters, thus the total DCG-FT metric value of malicious services in the top-*k* recommendation list is the lower, the better. Therefore, the above experiment and analysis show that our IRLT model greatly outperforms the state-of-the-art CGLT model in terms of the robustness against ballot stuffing attack.

#### Bad Mouthing

**Fig 5:**
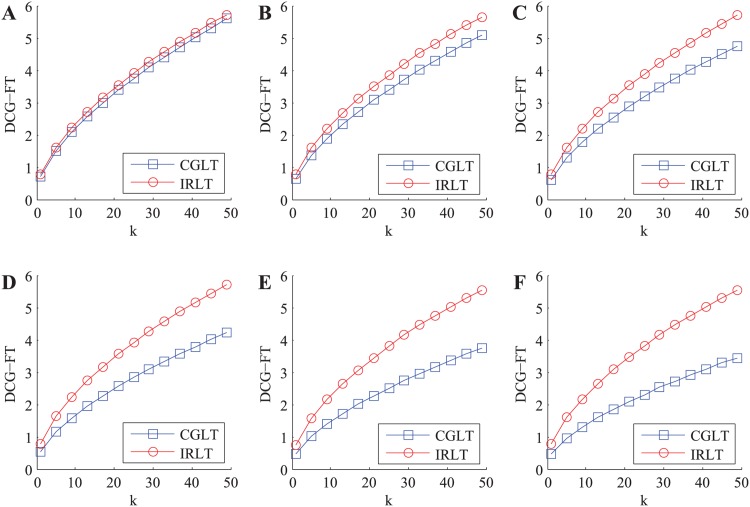
The variation of total DCG-FT metric value of honest services with *k* in malicious environment with bad mouthing attack. A: PCSC = 0%. B: PCSC = 20%. C: PCSC = 40%. D: PCSC = 60%. E: PCSC = 80%. F: PCSC = 100%.

Next, we compare the robustness of our IRLT model against bad mouthing attack with that of the CGLT model. In the bad mouthing attack, the collusive service consumers provide low rating scores to honest services in spite of their good performances. In each experiment, we vary PCSC and calculate the three kinds of metric values of honest services in the top-*k* recommendation list in each case, respectively. The experiment is also repeated 5000 times and the average outputs of DCG-FT metric are shown in Fig 5.

In the ideal case (i.e. PCSC = 0%, see Fig 5A), the variation curves of the total DCG-FT metric values of honest services in two kinds of trust models are very close to each other. With the increase of PCSC, the curve growth in the CGLT model gets slower and slower while that in our IRLT model remains about the same, thus the gap of two variation curves gradually grows. In the extreme case (i.e. PCSC = 100%, see Fig 5F), the gap of two variation curves is up to the maximum value and the total DCG-FT metric value of honest services in our IRLT model is greatly higher than that in the CGLT model.

It is well known that honest services will bring benefit to service requesters, thus the total DCG-FT metric value of honest services in the top-*k* recommendation list is the higher, the better. As a result, the above experiment and analysis demonstrate that our IRLT model is vastly superior to the outstanding CGLT model in terms of the robustness against bad mouthing attack.

#### Ballot Stuffing and Bad Mouthing

**Fig 6:**
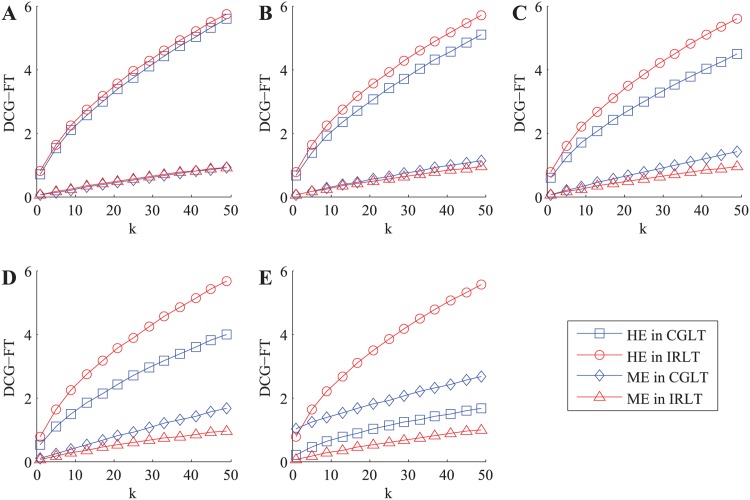
The variations of total DCG-FT metric values of honest services (HS) and malicious services (MS) with *k* in malicious environment with ballot stuffing and bad mouthing attacks. A: PCSC = 0%. B: PCSC = 25%. C: PCSC = 50%. D: PCSC = 75%. E: PCSC = 100%.

In this part, we verify the recommendation performance of our IRLT model in malicious environment with both ballot stuffing and bad mouthing attacks, in which the collusive service consumers not only provide high rating scores to malicious services but also provide low ones to honest services. In each experiment, we vary PCSC and calculate the three kinds of metric values of honest services and malicious services in the top-*k* recommendation list in each case, respectively. The experiment is also repeated 5000 times and the average results of DCG-FT metric are shown in Fig 6.

In the ideal case (i.e. PCSC = 0%, see Fig 6A), the variation curves of the total DCG-FT metric values of both honest services and malicious services in our IRLT model approximately accord with those in the CGLT model, respectively. With the increase of PCSC, the curve growth of honest services in the CGLT model gets slower and slower and that of malicious services becomes faster and faster, while in our IRLT model the variation curves of both honest services and malicious services are nearly unchanged. In the extreme case (i.e. PCSC = 100%, see Fig 6E), the total DCG-FT metric value of malicious services in the CGLT model even exceeds that of honest services, which indicates that the collusive service consumers succeed in manipulating the trust values of both honest services and malicious services in this case. While in our CGLT model, the total DCG-FT metric value of honest services is apparently higher than that of malicious services. Furthermore, the total DCG-FT metric value of honest services in our IRLT model is greatly higher than that in the CGLT model, while the total DCG-FT metric value of malicious services in our IRLT model falls significantly below that in the CGLT model.

As we mentioned earlier, honest services will bring benefit and malicious services mean risk for service requesters, so the total DCG-FT metric value of honest services in the top-*k* recommendation list is the higher, the better, and that of malicious services is the lower, the better. Therefore, the above experiment and analysis show that our IRLT model has better recommendation performance than the excellent CGLT model in malicious environment with both ballot stuffing and bad mouthing attacks.

## Conclusion

In this work, we have proposed a novel IRLT model, which includes four modules, for TSR in S-SNs. In SRI module, a multi-QoS based filtering method is proposed to select out all the candidate services. In LTTE module, a MLT evaluation algorithm is presented to identify all the trustworthy service witnesses and only their service ratings are considered. In RTE module, the opinions of all the service witnesses are considered through filtering by the results of LTTE module to ease unfair rating attacks. Besides, the preference similarity and rating number are regarded as two important weights. In ATE module, the outputs of RTE and LTTE modules are integrated by weighted summation to calculate the finial trust values of candidate services as well as generate the top-*k* recommendation list. Furthermore, we have deployed a synthetic S-SN based on the famous Advogato dataset and adopted the well-known DCG metric to measure the service recommendation performance of our IRLT model through comparing to that of the state-of-the-art CGLT model. The results of experiments and analysis show that our IRLT model has slightly better service recommendation quality than the CGLT model in honest environment and greatly outperforms the CGLT model in terms of the robustness against two kinds of unfair rating attacks.
